# Diabetes-Resistant NOR Mice Are More Severely Affected by Streptozotocin Compared to the Diabetes-Prone NOD Mice: Correlations with Liver and Kidney GLUT2 Expressions

**DOI:** 10.1155/2015/450128

**Published:** 2015-01-28

**Authors:** S. Kahraman, C. Aydin, G. O. Elpek, E. Dirice, A. D. Sanlioglu

**Affiliations:** ^1^Center for Gene and Cell Therapy, Akdeniz University, 07058 Antalya, Turkey; ^2^Center for Genetic Diagnosis, Akdeniz University, 07058 Antalya, Turkey; ^3^Department of Pathology, Akdeniz University Faculty of Medicine, 07058 Antalya, Turkey; ^4^Section of Islet Cell and Regenerative Medicine, Joslin Diabetes Center, Harvard Medical School, Boston, MA 02215, USA

## Abstract

Nonobese Diabetic (NOD) mice are susceptible strains for Type 1 diabetes development, and Nonobese Diabetes-Resistant (NOR) mice are defined as suitable controls for NOD mice in non-MHC-related research. Diabetes is often accelerated in NOD mice via Streptozotocin (STZ). STZ is taken inside cells via GLUT2 transmembrane carrier proteins, the major glucose transporter isoforms in pancreatic beta cells, liver, kidneys, and the small intestine. We observed severe adverse effects in NOR mice treated with STZ compared to NOD mice that were made diabetic with a similar dose. We suggested that the underlying mechanism could be differential GLUT2 expressions in pancreatic beta cells, yet immunofluorescent and immunohistochemical studies revealed similar GLUT2 expression levels. We also detected GLUT2 expression profiles in NOD and NOR hepatic and renal tissues by western blot analysis and observed considerably higher GLUT2 expression levels in liver and kidney tissues of NOR mice. Although beta cell GLUT2 expression levels are frequently evaluated as a marker predicting STZ sensitivity in animal models, we report here very different diabetic responses to STZ in two different animal strains, in spite of similar initial GLUT2 expressions in beta cells. Furthermore, use of NOR mice in STZ-mediated experimental diabetes settings should be considered accordingly.

## 1. Introduction

Nonobese Diabetic (NOD) mice are the most frequently preferred animal models in research related to type 1 diabetes (T1D) and other autoimmune conditions [1, 2]. Selection of appropriate controls for NOD mice depends on the type and aim of research. Nonobese Diabetes-Resistant (NOR) mice are defined by the producer Jackson Lab as suitable control strains for NOD mice in non-MHC-related studies. NOR/LtJ mice produced by Jackson Lab are thought to have originated from the NOD/LtJ strain through a genetic contamination with C57BLKS/J, where limited regions of the NOD/LtJ genome have been replaced by genome from the C57BLKS/J strain. While sharing the same MHC as NOD mice, NOR mice display a stronger T cell function compared to NOD mice, and islet inflammation in these mice does not progress beyond peri-insulitis [3, 4].

Spontaneous diabetes development takes approximately 30 weeks in NOD mice with a requirement for pathogen-free conditions. Thus, development of diabetes is frequently accelerated in NOD mice via agents such as Streptozotocin (STZ) and Cyclophosphamide (CY). While CY exerts its primary effect on CD4^+^CD25^+^ Treg cells [5], STZ is internalized through GLUT2 glucose transporters which are located exclusively on beta cells in the pancreatic setting, and also in liver, kidney, and small intestine cells. Accordingly, GLUT2 knockout mice are not susceptible to STZ [6]. STZ is an antibiotic produced by* Streptomyces achromogenes* and is also used as an FDA-approved drug in the metastatic cancer of pancreatic islets cells. It inhibits glucose oxidation and glucose-induced insulin secretion in beta cells via nitric oxide production, alkylation, and DNA fragmentation [7–10]. DNA fragmentation is followed by poly(ADP-ribose) activation and a sustained reduction in cellular NAD^+^ levels that induces cell death [6]. STZ can directly methylate nuclear and mitochondrial DNA and increases FasL expression.

Glucose transporter 2 (GLUT2) is a transmembrane carrier protein which provides passive glucose transport through the cell membrane. While being a member of facilitative glucose transporters family which uptake and release glucose and other certain sugars, GLUT2 is unique in accepting fructose as a substrate and also in its ability to act as a glucose sensor [11]. Thus, it plays an important role in glucose homeostasis, functioning as a part of the glucose sensory mechanism in pancreatic beta cells [12]. GLUT2 is expressed primarily in pancreatic beta cells and liver cells, as well as in absorptive epithelial cells such as the basolateral membrane of the kidney proximal tubules, and the small intestine [13]. GLUT2 is known to act as the main glucose transporter and sensor in rodent islets. Although claimed not to be the primary glucose transporter in human beta cells, SNPs in GLUT2 gene was found to predict conversion to diabetes from impaired glucose tolerance [14–16].

GLUT2 expression levels in pancreatic beta cells are defined as a marker for degree of susceptibility to STZ in various animal models. For instance, Old World Monkeys have lower levels of GLUT2 expression in beta cells, which is reflected as resistance to STZ. On the other hand, New World Monkeys with higher GLUT2 expression in pancreatic beta cells are fairly susceptible to the diabetogenic effects of STZ [17]. STZ leads to kidney and liver toxicity as well as beta cell damage [18].

In a previous study by our group, where we accelerated T1D in NOD mice by STZ and CY applications, NOR mice were put through similar applications as a control strain [19]. However, NOR mice displayed a considerably higher sensitivity against STZ compared to the NOD mice. While no unexpected deaths were observed in NOD mice during the 14-day follow-up after STZ application, sudden deaths were evident in NOR mice starting from day six. CY, which exerts its diabetogenic effect through regulator T cell suppression, did not have any diabetic/toxic effects on NOR mice. We hypothesized that the strong effect of STZ in these mice could be due to a species-specific difference, most likely related with high GLUT2 receptor expression in pancreatic beta cells. Thus we investigated* in situ* GLUT2 expression levels in pancreatic tissues of NOD and NOR mice prior to and following STZ application, while evaluating pancreatic islet compositions in these mice through various stages of disease. In addition, GLUT2 expression levels in liver and kidney tissues of the two strains were compared by western blot protein analysis. 

## 2. Materials and Methods

### 2.1. Animal Tissues

Animal tissues used in this study (paraffin-embedded pancreata and frozen liver and kidneys) belong to female NOD and NOR mice that were purchased from the Jackson Laboratory, USA, for an earlier study [19]. All animals had received a single dose of 150 mg/kg STZ at 10 weeks of age.

### 2.2. Ethics Statement

All animal works in the previous study [19], which provided the frozen and paraffin-embedded materials for the present study, were conducted under the approval of the Institutional Animal Care and Use Ethics Committee of Akdeniz University (Permit number 07-09/01) and in accordance with the Helsinki Declaration guidelines. Applied procedures included STZ injections, anesthesia, and tissue extractions.

### 2.3. Immunofluorescence Stainings and Analysis

For immunofluorescence staining, slides were first kept at 60°C for two hours. After xylol and alcohol series, heat-mediated antigen retrieval was performed in 10 mM sodium citrate buffer (pH 6.0). Donkey serum (5%) was applied for 1 hour at room temperature (RT) to prevent nonspecific stainings. Overnight incubation at 4°C by primary antibodies was done in following dilutions: guinea pig anti-insulin at 1/100 dilution (Abcam, ab7842), rabbit anti-glucagon at 1/50 dilution (Abcam, ab18461), and goat anti-GLUT2 at 1/50 dilution (Abcam, ab111117). Next day after PBS wash series, secondary antibodies were applied for 1 hour at RT at 1/200 dilution: anti-guinea pig CY2 (Jackson Immunoresearch, 706-226-148), anti-rabbit Texas Red (Santa Cruz, sc-2784), and anti-goat Texas Red (Abcam, ab7123). PBS wash series were then followed by application of DAPI-containing mounting medium.

ImageJ analysis program was used for quantitative analysis of stainings. For GLUT2 stainings, GLUT2 fluorescence rate was normalized to the surface area of the insulin-positive cells. Colored pictures taken from dual-stained islets were converted into gray-toned images. Insulin-stained areas were defined in individual islets, and the corresponding surface areas were calculated. These defined areas were then merged on the GLUT2-stained areas, and the fluorescence intensities were determined. Dividing the measured fluorescence intensities into surface area values of the insulin-stained cells gave us the “mean fluorescence intensities” [20]. Three different pancreata and 5 to 25 islets from each of the pancreata were analysed.

### 2.4. Identification of Alpha and Beta Cell Ratios

Alpha and beta cell ratios were determined by counting insulin and glucagon-stained cells in islets following dual immunofluorescence stainings. Three different pancreata were examined to reach average values in each study group, with 5 to 25 islets evaluated in each pancreas.

### 2.5. Immunohistochemical Stainings and Analysis

Sections of 5 *μ*m thickness were deparaffinized through xylol and alcohol series. Heat-mediated antigen retrieval application in 10 mM sodium citrate buffer (pH 6.0) was followed by quenching of endogenous peroxidase activity through hydrogen peroxide block treatment for 30 minutes at RT. Ultra V blocking was performed for 5 minutes at RT for inhibition of nonspecific stainings. After removal of blocking solution, sections were incubated with rabbit-derived primary antibody against GLUT2 receptor at 4°C overnight, at a 1/50 antibody dilution (Abcam, ab104622). Next day, tissues were washed in PBS series and kept in HRP-conjugated anti-rabbit secondary antibody (Abcam, ab6721), at a 1/200 dilution, for 1 hour at RT. PBS wash series were followed by DAB chromogen incubation for 1 min. After 20 seconds of hematoxylin staining of the cell nuclei, alcohol and xylol series were applied. Slides were mounted with Entellan (Merck, 7961).

The stained slides were blind-evaluated by a pathologist (GOE) who had no prior knowledge on tissue origins. The scoring system is based on both intensity and distribution (percentage of the positively stained cells) of GLUT2 stainings. Positive cells counted at 400x magnification were evaluated relative to the total number of cells. Intensity of stainings was classified as (0) negative; (1) weak; (2) moderate; and (3) strong. Marker distribution was scored as (0) less than 10%, (1) between 10% and 40%, (2) between 40% and 70%, and (3) for more than 70% of the cells stained positive. The sum of the intensity and marker distribution scores gave the final staining score.

### 2.6. Western Blotting

Frozen kidneys and livers from NOD and NOR mice were homogenized in lysis buffer (pH 7.4) containing 100 mM NaF, 50 mM Hepes, 150 mM NaCl, 10% Glycerol, 1.2% Triton X, 1 mM MgCl_2_, 1 mM EDTA, 1 mM Na_3_VO_4_, and protease inhibitor cocktail (Roche, 11836145001). Total protein concentration was determined by Bradford Protein Assay (Bio-Rad). Lysates (50 *μ*g protein) were run in 8% SDS-PAGE and transferred to PVDF membrane (Millipore). Membranes were blocked for 10 minutes at room temperature with 5% milk and were incubated overnight at 4°C with antibodies against GLUT2 (1 : 1,000, sc-9117; Santa Cruz Biotechnology) or *β*-actin (1 : 1,000, sc-81178; Santa Cruz Biotechnology). After three washing series (10 minutes), the membranes were incubated for 1 hour at RT with antibodies against rabbit IgG-HRP conjugate (1; 2000, #170-6515, Bio-Rad) or mouse IgG-HRP conjugate (1; 2000, #170-6516, Bio-Rad). After three 10-minute washes, signals were visualized via ECL (Roche) and quantified using ImageQuant version 5.1 software.

### 2.7. Statistical Analysis

Immunohistochemistry results were statistically analysed by Prism GraphPad software (version 5.0a.128). Normality tests were carried out by Shapiro-Wilk method, and Mann-Whitney *U* test was used for comparisons of the scores between two independent groups. Statistics for the immunofluorescent results were done via Student's *t*-test. Statistical significance was considered at 5% probability level (*P* < 0.05).

## 3. Results

### 3.1. Structural Alterations in the Pancreatic Islets and Quantitative Changes in the Islet Cell Contents of NOD and NOR Mice following STZ Application

**Figure 1:**
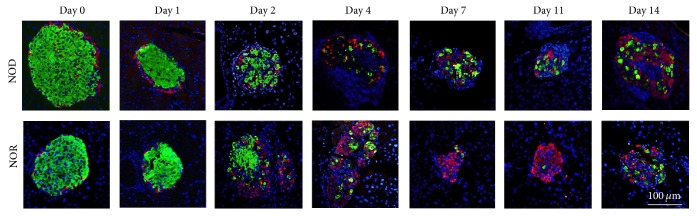
Changes in pancreatic islet content following STZ treatment. Representative images showing pancreatic islet contents in NOD (upper panel) and NOR mice (lower panel) before and after STZ application. Pancreatic tissues were stained with anti-insulin (green) and anti-glucagon (red) antibodies, and counterstaining was done with DAPI nuclear stain. Pictures represent islet images of pancreatic tissues isolated from animals sacrificed on day 0 (before STZ injection) and on days 1, 2, 4, 7, 11, and 14, after 150 mg/kg STZ injection (*n* = 3 for each time point).

**Figure 2:**
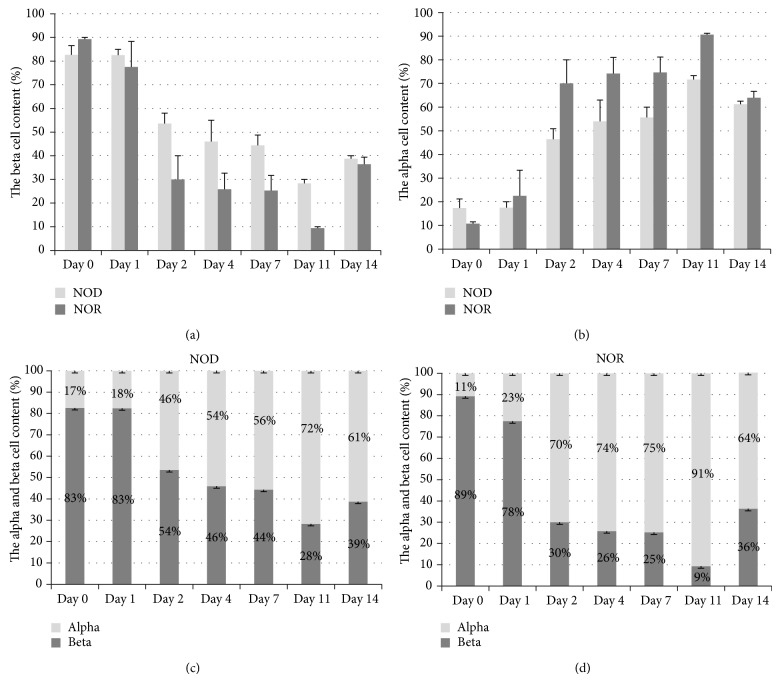
Quantitative analysis of alpha and beta cell contents in pancreatic islets of NOD and NOR mice following STZ injection. Upper panel displays changes in beta (a) and alpha (b) cell percentages within the islets compared in NOD (lighter bars) and NOR mice (darker bars). In the lower panel, progressive alterations in comparative proportions of alpha and beta cell contents are shown in NOD (c) and in NOR mice (d). ^*^ *P* < 0.05, NOD versus NOR (Student's *t*-test). Error bars represent ± SEM (*n* = 3 for each time point).

Following a single dose of 150 mg/kg STZ injection, the pancreatic islet morphology changed gradually in both NOD and NOR mice during the 14 days of follow-up. Beta cell numbers decreased in both strains, with a faster rate in NOR mice (Figure 1). We observed that NOR mice had nearly half the beta cell content compared to that of NOD mice at day 2 and less number of beta cells at all points after that. Proportion of alpha cells increased gradually in both strains. Alpha and beta cell percentages in each test point are shown in Figure 2. 

### 3.2. Expression Levels of GLUT2 in NOD and NOR Pancreatic Islets before and after STZ Application

**Figure 3:**
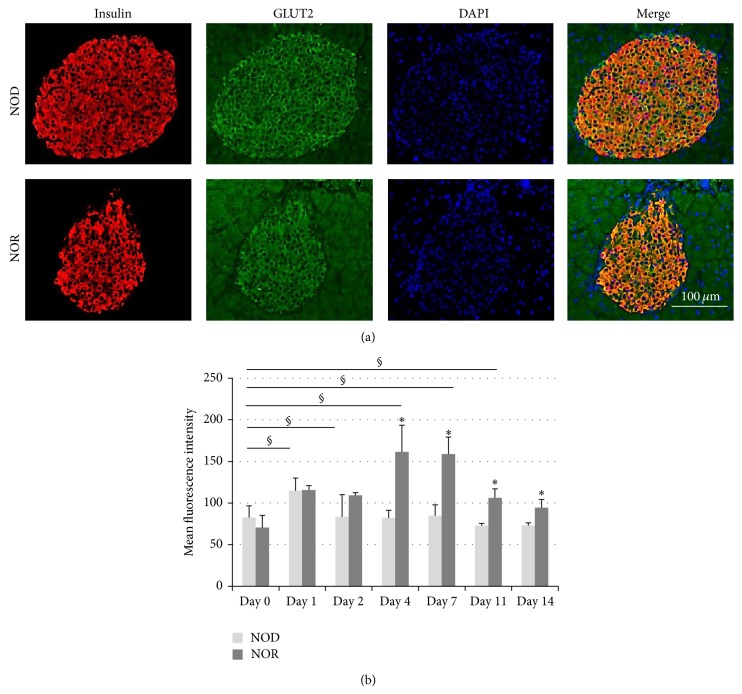
GLUT2 expression levels in the pancreatic beta cells of NOD and NOR mice. (a) Representative images of dual stainings for insulin (red) and GLUT2 expressions (green) in the presence of DAPI counter-staining (blue), before STZ application (day 0). (b) Mean fluorescence intensities for GLUT2 expression in NOD (lighter bars) and NOR islet beta cells (darker bars) on days 0, 1, 2, 4, 7, 11, and 14 of STZ injection. Five to twenty islets per animal were analysed via ImageJ program (*n* = 3/group). ^*^ *P* < 0.05, significance between values observed in NOD versus NOR mice; ^§^ *P* < 0.05, significance between values detected in NOR mice before STZ injection and at different time points after treatment (Student's *t*-test).

**Figure 4:**
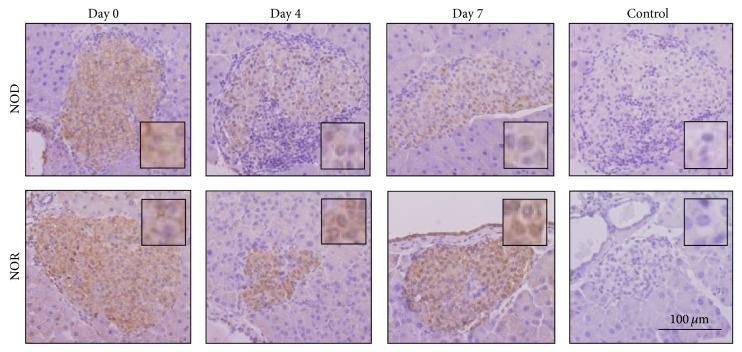
GLUT2 immunohistochemistry stainings in NOD and NOR islets. Representative islet images are shown demonstrating GLUT2 expression levels prior to (day 0) and after STZ application (days 4 and 7). Hematoxylin counterstaining was performed following anti-GLUT2 antibody application. Tissues depicted as “control” were treated with secondary antibody only.

Pancreatic islets of NOD and NOR mice were dually stained for insulin and GLUT2 expressions prior to and following STZ injection, and immunofluorescence intensities were analysed. Similar GLUT2 expression levels were evident in both mice strains at day 0 (Figure 3). Differences in islet GLUT2 expressions between NOD and NOR mice following STZ application were comparatively analysed. Furthermore, progressive changes in islet GLUT2 expression levels after STZ injection were evaluated separately in each of the mice in comparison to the day 0 values of the corresponding strain. According to our results, GLUT2 levels did not change significantly in islets of NOD mice throughout the 14-day follow-up, while it was elevated in NOR mice starting from day 4 of injection. GLUT 2 expression levels in NOR mice islets were significantly higher compared to NODs, between days 4 and 14. GLUT2 expressions were maintained significantly higher in NOR mice after STZ injection, in comparison to day 0 values (Figure 3(b)). Immunohistochemical stainings revealed similar results (Figure 4). 

### 3.3. GLUT2 Expression Levels in Livers and Kidneys of NOD and NOR Mice Prior to STZ Application

**Figure 5:**
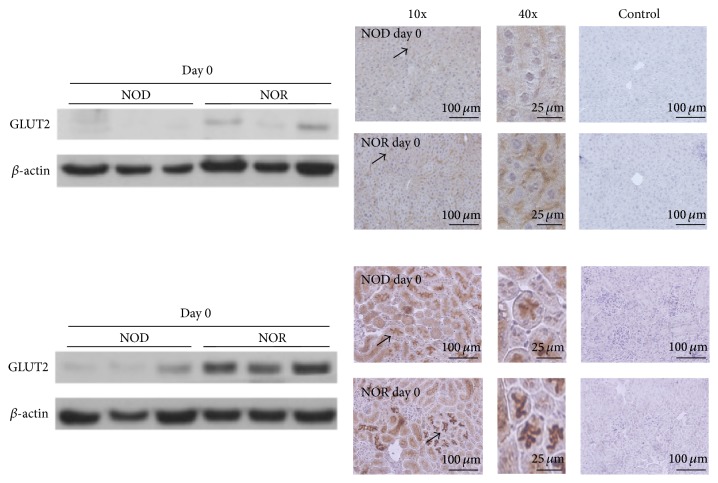
Western blot analysis and immunohistochemical stainings of GLUT2 expressions in the livers and kidneys of non-STZ treated NOD and NOR mice. Representative images are shown demonstrating GLUT2 expression levels in livers (upper panel) and kidneys (lower panel) of NOD and NOR mice via western blot (left) and immunohistochemical stainings (right), prior to STZ application (day 0). Hematoxylin counterstaining was performed following anti-GLUT2 antibody application. Immunohistochemistry images show the corresponding tissues at 10x magnification on the left side, and at 40x magnification in the middle depictions. Arrows are placed for comparison of the staining intensities. Control tissues, treated with secondary antibody only, are shown at right.

Lysates obtained from frozen kidneys and livers of the non-STZ treated NOD and NOR mice (day 0 tissues) were used for western blot analysis of GLUT2 expressions (Figure 5). Scarce levels of GLUT2 were detected in the livers of 10-week-old, non-STZ treated female NOD mice. The sex- and age-matched NOR mice, on the other hand, displayed higher levels of GLUT2 in liver. This result was confirmed by immunohistochemical stainings on the same tissues following paraffin embedding (Figure 5). GLUT2 levels in the kidneys of the non-STZ treated NOR mice were substantially higher compared to the NODs. Immunohistochemical stainings revealed similar results.

## 4. Discussion

Streptozotocin (STZ) is a glucose analogue frequently used as a diabetic agent in various animal models. We previously observed strong adverse effects caused by 150 mg/kg STZ injection in 10-week-old female Nonobese Diabetes-Resistant (NOR) mice that were intended to be used as age- and sex-matched controls for Nonobese Diabetic (NOD) mice [19]. To investigate the underlying mechanism, we examined pancreas, liver, and kidney tissues of the animals for possible differences in GLUT2 expression levels between the two mice strains.

As expected, STZ injection triggered a decrease in pancreatic beta cell count, in both NOD and NOR mice [21, 22]. Interestingly, NOR mice displayed a faster decrease in beta cell numbers and a wider difference between the alpha and beta cell percentages within the islets, at most time points chosen for analysis (Figure 2). Yet in spite of fast reductions in beta cell numbers, GLUT2 expression levels significantly increased gradually in the islets of NOR mice, unlike those of the NOD mice (Figure 3). Higher blood sugar levels were also evident in these mice in periodic measurements, compared to the NOD mice [19]. As GLUT2 expression is confined to beta cells in the islet setting, higher blood sugar concentrations due to STZ-induced beta cell failure is likely to be the mechanism responsible from the rise in GLUT2 expression levels. Glucose-stimulated upregulation of GLUT2 expression was shown in hepatocytes via direct effect on gene transcription and in rat proximal tubule brush border membrane [13, 23, 24]. In contrast, islets prepared from C57BL/6 male mice treated with multiple low doses of STZ displayed decreased GLUT2 expression levels [25]. Multiple low doses of STZ are related to induction of autoimmune diabetes. We applied a single high dose of STZ to both NOD and NOR mice, which rather caused quick and sharp rises in blood sugar levels apparently due to more immediate beta cell destruction, thus to immediate high blood sugar levels and presumably the increased GLUT2 expression levels. It is also noteworthy that we observed no rise in GLUT2 expression levels in the pancreatic beta cells of the T1D-prone NOD mice, while GLUT2 levels were significantly raised in the pancreatic beta cells of the T1D resistant NOR mice.

In general, NOR mice had higher blood sugar levels and were affected more adversely from STZ application compared to the NODs. This may reflect a somewhat higher exposure of NOR mice to STZ. We suggested that a possible explanation might involve higher GLUT2 expression levels in the islets of NOR mice before STZ application. However, we found no significant differences in islet GLUT2 expression levels measured prior to STZ injection in NOD and NOR mice (Figure 3).

STZ is also known to affect liver and kidney tissues at varying degrees in different species. This effect is in accordance with localization of GLUT2 receptors primarily in pancreatic beta cells and hepatocytes, as well as in epithelial cells of the kidney [13]. As well known, kidneys reabsorb all the filtered glucose and thus play an important role in glucose homeostasis, to ensure sufficient energy for the fasting conditions. This mechanism is known to be destructed in the diabetic setting because hyperglycemia increases the expression and activity of GLUT2 receptors in the kidney proximal tubules [26]. STZ-mediated diabetes induction interferes with glucose transport in kidneys as well [27]. Furthermore, STZ-induced diabetes in rats is known to produce alterations in structures of hepatocytes and the hepatic functions, accompanied by impairment in glucose utilization in STZ-induced diabetes [28]. STZ induction in HepG2 liver cells led to an increase in oxidative stress markers, caused mitochondrial respiratory dysfunction, and resulted in limited induction of mitochondrial apoptotic pathways [29]. Increase in GLUT2 expression levels in a glucose concentration manner was reported in primary rat hepatocytes and hepatocyte cell lines [24, 30]. Thus higher GLUT2 expression levels in the liver and kidney tissues of the NOR mice compared to NODs before STZ application suggested higher initial exposures to STZ in these tissues and thus higher toxicity.

Differing degrees of GLUT2 expression levels in pancreatic, hepatic, and renal tissues, as well as between different species, appear to determine the intensity of the harm caused by STZ application at a given dose. For instance, common marmosets were reported as fairly resistant to any rise in blood sugar levels following STZ application due to low GLUT2 expression levels in pancreatic islets [17]. Furthermore, the toxic effects of STZ on livers and kidneys of common marmosets were reported to outweigh its diabetogenic effects. We observed that liver and kidney tissues of NOR mice displayed higher GLUT2 levels prior to STZ application, compared to the NOD mice (Figure 5). Although we do not have specific values to demonstrate the degree of hepatic and renal toxicity in the mice strains used in our study, NOR mice were in very poor health starting from day 4 of STZ application, with observable fatigue and lack of motion, most likely due to very high blood sugar levels (measured over 600 mg/dL).

In consequence, our results reveal more adverse effects in the diabetes-resistant NOR mice in response to injection of a single dose of (150 mg/kg) STZ, compared to the diabetes-prone NOD mice which received the same treatment. Yet we could not correlate this adverse reaction with differential GLUT2 levels in pancreatic islets of the two strains as would be expected. Instead, more severe diabetogenic effects caused by STZ in NOR mice compared to NODs are revealed by extremely high blood glucose levels throughout the follow-up, correlated with higher GLUT2 levels in liver and kidney tissues. As well known, STZ is a frequently used experimental diabetes-inducing agent in animal models, as well as an FDA-approved chemotherapeutic drug. Besides revealing that NOR mice should be carefully considered for studies involving relatively high doses of STZ, our results also refer to the significance of individual differences in GLUT2 expression levels in hepatic and renal tissues as determinants of STZ toxicity levels.
